# Development of organizational healing scale: validity and reliability study

**DOI:** 10.3389/fpsyg.2024.1491182

**Published:** 2025-01-07

**Authors:** İsmail Karsantık, Semih Çayak

**Affiliations:** ^1^Department of Educational Sciences, Çayeli Faculty of Education, Recep Tayyip Erdogan University, Rize, Türkiye; ^2^Department of Educational Sciences, Atatürk Faculty of Education, Marmara University, Istanbul, Türkiye

**Keywords:** healing, trauma, recovery, organizational healing, scale development

## Abstract

**Introduction:**

Adverse and destructive situations can have a significant and long-lasting impact on organizational members, resulting in considerable disruption to organizational functioning. The occurrence of negative or traumatic events in organizations can be attributed to a range of factors, including natural adversities, as well as intentional or human-induced crises. The concept of organizational healing encompasses both the processes that enable an organization to regain functionality following adversity and the strategies that facilitate enhanced performance in the period following trauma or harm. Recent advances have highlighted the topic of organizational healing, particularly in relation to how organizations can recover from significant traumatic events and return to their pre-disaster state. This study aimed to develop the Organizational Healing Scale by verifying its reliability and validity.

**Methods:**

The item pool for the organizational healing scale was developed with the objective of ensuring its applicability for researchers and participants by adhering to the principles of scientific rigor and practicality. In this context, an item pool of 32 items was created. To ensure construct validity, EFA and CFA were conducted, and for content and face validity, expert opinion was consulted. Validity was also ensured through convergent and discriminant validity. Reliability was tested using Cronbach's alpha coefficient value.

**Results:**

The two components (individual priority, organizational priority) identified through EFA as contributing to the construct validity of the scale were subsequently confirmed by CFA. The fit indices for the scale were at satisfactory level. The Cronbach Alpha coefficient value demonstrated that both components were reliable.

**Discussion:**

A review of the results indicated that the organizational healing scale is a valid and reliable instrument for measuring the healing levels of organizations with respect to the component under consideration.

## 1 Introduction

Organizations established to achieve a specific purpose (Aytaç, 2004) may occasionally be confronted with unforeseen and adverse circumstances while engaged in their regular operations. Such destructive and adverse situations can have a profound traumatic impact on organizational members and significantly disrupt organizational functioning. In order to proactively mitigate the risk of such adverse situations, organizations implement a range of measures. However, in the contemporary era, developments can occur rapidly and in a complex manner. Consequently, organizations may find themselves in a state of crisis and chaos (Powley, 2012). The aforementioned adverse situations, which have the potential to inflict significant trauma on organizations, may be precipitated by natural disasters such as earthquakes, fires, and floods, or they may result from human-induced crises, including robberies, armed attacks, terrorist acts, and severe financial crises (Demirtaş, 2000; Powley, 2013). Organizational healing denotes the capacity of organizations to resume their core activities following such significant crisis (Petterson, 1999). Similarly, as a healthy body becomes unwell and then recovers and regains its health, it is possible for organizations to recover from adverse situations and regain their normal order (Mitchell, 1996; Powley and Piderit, 2008). However, the internal structure and functioning of each organization is different, and thus, it is inevitable that there will be differences in the healing processes of organizations after crisis situations. For instance, as institutions dedicated to the dissemination of knowledge, schools are among the organizations that are most reliant on the contributions of human capital. The healing process in such organizations will differ from the healing of a bank or a company. Indeed, the media frequently depicts the traumatic effects of terrorist or violent acts that occur in schools in various global locations. However, it is challenging to eradicate the consequences of such incidents on students and educators, thereby facilitating organizational healing.

The concept of organizational healing is employed to describe the process by which organizations regain a state of wellbeing following a significant degree of damage. The term “healing” is commonly used in the field of health to describe the process of returning to a state of soundness, health, and wholeness after illness or harm (Powley and Cameron, 2006). However, healing is a process that extends beyond the mere healing of physical ailments (Criddle, 1993). In this sense, organizational healing refers to the work of repairing practices, routines, and structures in the face of disruption and strengthening organizational functioning through social relationships (Powley, 2013).

Organizational healing can be defined as a type of restoration that occurs during and after any event that disrupts organizational routines, structures, relationships between individuals, and an individual's life experience (Fazio and Fazio, 2005). In their examination of the healing of organizations that experienced traumatic events, Powley and Cameron (2006) identified four main enablers of the healing process through extensive research and long-term observations. These themes are as follows: “Reinforcing the priority of the individual,” which refers to the extent to which the organization demonstrates a commitment to the wellbeing, future, and career development of its members; “Fostering High Quality Connections,” which describes the degree to which organizational members intentionally cultivate robust personal relationships with one another; and “Strengthening a Family Culture,” which emphasizes the importance of a close-knit, supportive organizational culture. The term “Initiating Ceremonies and Rituals” refers to the degree to which rituals, ceremonies, and symbolism facilitate the restoration of stability, self-concept, and organizational identification among members. The term “close-knit, family-type organization” is defined as an organizational culture that fosters a sense of belonging and cohesion among individuals across boundaries.

The concept of organizational healing encompasses not only the processes through which organizations regain their functionality following adversity but also the ways in which they enhance their performance in the period following trauma or harm (Tedeschi and Calhoun, 1995). Shepherd's (2003) work on coping with business failure and losses contributes to an in-depth understanding of the learning processes of organizations. Shepherd (2003) posits that failures should not only be perceived as losses, but also as experiences that provide learning opportunities. This perspective is crucial for comprehending how organizations learn from past adverse experiences and how they undergo restructuring. In the aftermath of crises, organizations implement measures to foster the psychological and emotional wellbeing of their employees through a range of strategies. For example, post-crisis support programs may be designed to reduce employees' anxiety and increase their engagement (Cope, 2011). Furthermore, social support and network relationships play a critical role in organizations' healing processes. The sharing of experiences within social networks, where entrepreneurs and organizations can interact, can facilitate the healing process (Izquierdo and Buelens, 2008). In this sense, organizational healing is distinct from other related concepts in organizational sciences, including hardiness, resilience, and recovery. This is because resilience is an individual characteristic, attributed to managers or other organizational members, who are able to cope with setbacks and withstand difficult circumstances. It is not a process of repair and restoration. Furthermore, healing is distinguished from resilience. Resilience is a latent capacity inherent to both individuals and organizations prior to the occurrence of any traumatic event. Resilience can be defined as the capacity to recover from adverse events and to withstand disruptions (Sutcliffe and Vogus, 2003). In contrast, healing is an active process involving social interaction that occurs after a crisis in order to ensure the restoration of the organization. The concept of healing is closely related to, but conceptually distinct from, the concept of recovery. The term “recovery” is used to describe a long-term process that enables systems affected by trauma or injury to resume their normal routines and functions. It is important to note that recovery is fundamentally different from the process of healing in terms of its temporal dimension. Unlike healing, which is a relatively short-term process that begins immediately after the crisis or trauma occurs and is measured in hours, days, or weeks (Powley and Piderit, 2008), recovery is a long-term process that can span months, years, or even decades. In the context of organizational studies, the term “organizational healing” is used to describe the process by which an organization rebounds and reorganizes following a traumatic event. This concept is distinct from that of organizational resilience, which pertains to an organization's capacity to withstand and recover from such events (Luthans et al., 2006; Tugade and Fredrickson, 2004). The primary distinction between these two concepts lies in their respective focal points and operational mechanisms. The objective of organizational healing is to surmount the consequences of trauma and reinstate the psychological and emotional wellbeing of employees. These processes encompass the implementation of strategies designed to foster trust among employees, enhance motivation, and reinforce the organizational culture. For example, post-crisis support programs may seek to mitigate employee anxiety and enhance engagement (Brewin et al., 2009). In contrast, organizational resilience can be defined as an organization's capacity to withstand challenges and demonstrate adaptability in the face of adversity. The concept of resilience is concerned with the capacity of an organization to respond effectively to unforeseen circumstances and to maintain its functionality throughout this process. A resilient organization demonstrates the capacity to rapidly adapt and alter its strategic orientation in response to changing circumstances and uncertainty. This contributes to the organization's ability to not only survive but also thrive in the post-crisis period (Berkman and Glass, 2000; Masten, 2001). Consequently, while recovery focuses on the aftermath of a specific crisis, resilience emphasizes the overall flexibility and adaptability of organizations. Both concepts are critical in enhancing the sustainability of organizations and ensuring effective crisis management.

**Figure 1:**
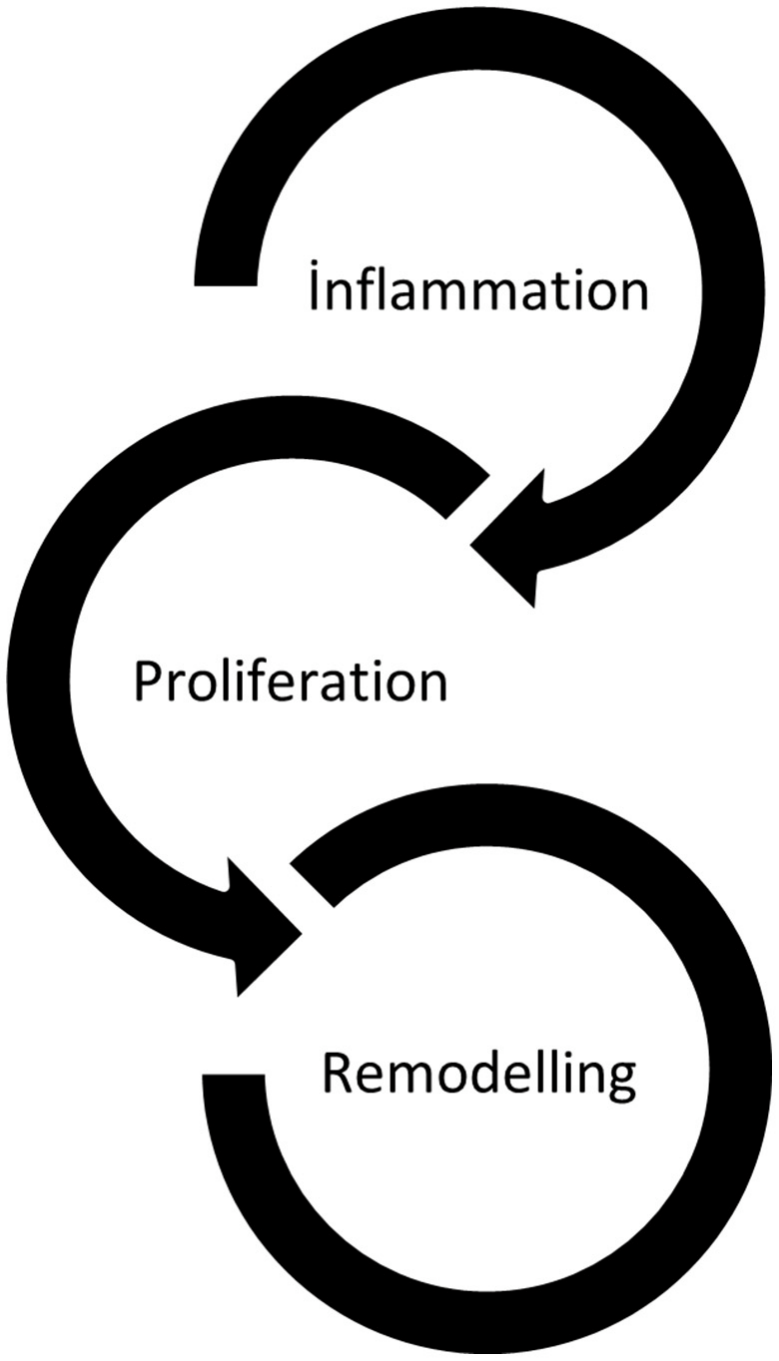
Model of organizational healing (Powley and Piderit, 2008).

Powley and Piderit (2008) argue that the wound healing process in medicine can be conceptualized as a rich metaphor for exploring organizational healing. Schilling (1968) research on wound healing identifies three fundamental stages in the healing process: inflammation, proliferation, and maturation. Based on these stages, Powley and Piderit (2008) propose a theoretical model of organizational healing that includes three healing stages and six key facilitators. These are: inflammation, which involves prioritizing the individual in need of immediate care and addressing the potential for blame; proliferation, which entails fostering high-quality connections and improvising on routines; and remodeling, which involves strengthening a family culture and initiating ceremonies and rituals (Figure 1).

Powley (2012) delineates the inflammation stage as the pivotal actions undertaken by both leaders and members to safeguard themselves from further trauma. The objective of this stage, as outlined by Powley, is to “secure and ensure the collective wellbeing of the organization's members.” For organizations, the inflammation stage necessitates an internal triage process. Those individuals who are most in need of care are accorded the highest priority and are attended to in the shortest possible time. In this phase, members of the organization endeavor to ascertain the root causes of the original trauma. However, in the inflammation phase, members of the organization are particularly advised to eschew mutual recrimination. In the proliferation phase, members of the organization endeavor to identify and foster connections between each other. The shared experience of organizational members provides an opportunity for them to gain insight into each other's perspectives and establish connections that may not be possible with individuals outside the organization. This can only occur when members of the organization facilitate a sense of comfort and belonging for others within the organizational structure. In the remodeling phase, the organizational culture is reinforced through the sharing of a collective history and the healing process. The group has emerged from the healing process in a robust state, and the organization has become more resilient as a result of shared experiences. Consequently, shared experiences engender a more profound sense of community, and the rituals of the group evolve to reflect this new phase (Powley and Piderit, 2008). Indeed, Powley (2012) asserts that the objective of the reshaping phase is not to revert to a previous state, but to transition to a productive organizational structure that is better equipped to withstand future harms.

In light of the aforementioned information, organizational healing can be defined as the process of addressing and resolving issues that have the potential to cause harm or dysfunction within an organization (Johnson, 2023). In this context, organizational healing involves the addressing of conflicts, problems, and other issues between employees or departments, as well as the examination of the effects of organizational change. The promotion of organizational healing can be achieved through the implementation of effective communication strategies, the establishment of a supportive work environment, and a focus on the wellbeing of employees (Powley, 2012). Consequently, as in all managerial processes, organizational leaders assume significant responsibilities in the healing process (Wallace and Witherspoon, 1998). Leaders who support the healing process at all stages of healing consciously prioritize the establishment of connections between members of the organization and internal and external stakeholders. It is therefore anticipated that their remedial leadership behaviors will effectively diagnose, treat, healing and eliminate organizational problems (Wallace, 2001). In this respect, leaders are expected to implement certain key behaviors to restore a healthy work environment (Byrd-Poller et al., 2017).

### 1.1 Purpose of the research

The topic of organizational healing has recently been a prominent area of study in the field of management literature, as it pertains to the ability of organizations to return to their pre-disaster state following a significant traumatic event. However, an examination of the studies on this subject reveals that the majority of them are literature reviews or case studies (Powley and Cameron, 2006; Powley and Piderit, 2008; Powley and Taylor, 2010; Powley, 2012, 2013; Wallace, 2001). It is therefore necessary to conduct further research on organizational healing using quantitative research methods. However, prior to this, a scale for determining the healing levels of organizations must first be developed. Utilizing quantitative research techniques, the present study was undertaken with the objective of developing a valid and reliable scale for the assessment of organizational healing following traumatic events or major crisis situations.

## 2 Materials and methods

### 2.1 Sample of the study

A simple random sampling method was employed to identify the participants. This sampling method is distinguished by the fact that the units in the population are equal and independent in the selection of the sample (Büyüköztürk et al., 2020; Karasar, 2012, p. 113). The research sample comprises educators employed by educational institutions affiliated with the Ministry of National Education in Turkey.

As seen in the Table 1, 318 (72.1%) of the individuals who participated in the EFA study were female and 123 (27.9%) were male. A total of 61 (13.8%) individuals aged 22–29, 158 (35.8%) individuals aged 30–39, 180 (40.8%) individuals aged 40–49, and 42 (9.5%) individuals aged 50 and over participated in the study. When the professional experience variable is analyzed, it is seen that the number of participants with 6–10 years of experience is 99 (22.4%), 11–15 years of experience is 105 (23.8%), 16–20 years of experience is 59 (13.4%), 21–25 years of experience is 48 (10.9%), and 25 years and above is 41 (9.3%). The distribution of educational organizations according to level of education indicates that 4 (20%) and 5 (25%) are pre-school and first level (grades 1–4) respectively, 5 (25%) are second level (grades 5–8) and 6 (30%) are third level (grades 9–12). The organizations were subsequently categorized according to the number of students enrolled. In the context of EFA, the number of students in the 0–300 and 901+ ranges each constituted 3 (15%) of the total, while the number of students in the 301–600 and 601–900 ranges each made up 7 (35%). The teaching staff numbers were also evaluated, with organizations having 0–20, 21–40, and 41–60 staff members accounting for 5 (25%), 9 (45%), and 6 (30%), respectively.

**Table 1:** Demographic characteristics of the participants.

**Variable**	**EFA**	***n***	**%**	**CFA**	***n***	**%**
Gender	Female	318	72.1	Female	269	73.5
	Male	123	27.9	Male	97	26.5
Age	22–29 years old	61	13.8	22–29 years old	45	12.3
	30–39 years old	158	35.8	30–39 years old	124	33.9
	40–49 years old	180	40.8	40–49 years old	158	43.2
	50 years old and over	42	9.5	50 years old and over	39	10.7
Professional experience	0–5 years	88	20.2	0–5 years	67	18.3
	6–10 years	99	22.4	6–10 years	87	23.8
	11–15 years	105	23.8	11–15 years	82	22.4
	16–20 years	59	13.4	16–20 years	51	13.9
	21–25 years	48	10.9	21–25 years	41	11.2
	25 years and over	41	9.3	25 years and over	38	10.4
The number of educational organizations (classified according to level)	Pre-school	4	20.0	Pre-school	5	25.0
	First level (1st, 2nd, 3rd, and 4th grade)	5	25.0	First level (1st, 2nd, 3rd, and 4th grade)	4	20.0
	Second stage (5th, 6th, 7th, and 8th grade)	5	25.0	Second stage (5th, 6th, 7th, and 8th grade)	6	30.0
	Third level (9th, 10th, 11th, and 12th grade)	6	30.0	Third level (9th, 10th, 11th, and 12th grade)	5	25.0
The number of educational organizations, classified by the number of students enrolled	0–300	3	15.0	0–300	5	25.0
	301–600	7	35.0	301–600	5	25.0
	601–900	7	35.0	601–900	8	40.0
	901+	3	15.0	901+	2	10.0
The current number of teaching staff employed at educational organization	0–20	5	25.0	0–20	6	30.0
	21–40	9	45.0	21–40	8	40.0
	41–60	6	30.0	41–60	6	30.0

Of the individuals who participated in the CFA study, 269 (73.5%) were female and 97 (26.5%) were male. A total of 45 (12.3%) individuals aged 22–29, 124 (33.9%) individuals aged 30–39, 158 (43.2%) individuals aged 40–49, and 39 (10.7%) individuals aged 50 and over participated in the study. When the professional experience variable is analyzed, it is seen that the number of participants with 6–10 years of experience is 87 (23.8%), 11–15 years of experience is 82 (22.4%), 16–20 years of experience is 51 (13.9%), 21–25 years of experience is 41 (11.2%), and 25 years and over is 38 (10.4%). The distribution of educational organizations according to level of education indicates that 5 (25%) and 4 (20%) are pre-school and first level (grades 1–4), respectively, 6 (30%) are second level (grades 5–8) and 5 (25%) are third level (grades 9–12). The organizations were also subsequently categorized according to the number of students enrolled. In the sample of CFA, the number of students in the 0–300 students were 5 (25%), 301–600 students were 5 (25%), 601–900 students were 8 (40%), and 901+ students were 2 (10%). The teaching staff numbers were also evaluated, with organizations having 0–20, 21–40, and 41–60 staff members accounting for 6 (30%), 8 (40%), and 6 (30%), respectively. Furthermore, all of the municipal entities in which the educational institutions and the teaching personnel involved in the research are situated are within the middle-income category (SEDI, 2022).

To prepare the dataset for analysis, the assumptions of exploratory factor analysis (sample size, homogeneity, linearity, multicollinearity) were first conducted. In the context of handling missing data, one effective approach is to utilize a method of imputation that replaces the missing values with plausible estimates that have been derived from the observed data. This approach is based on the premise that missing data can be filled in with statistically reasonable values, thus ensuring the overall integrity of the dataset is maintained. The most commonly utilized techniques for this type of imputation are mean, median, and mode imputation, through which missing values are replaced with the average, median, or most frequent value observed in the dataset, respectively (Gautam and Latifi, 2023). In this study, the mean values were assigned in the data set where the missing data was present. Data from 441 participants were used for the exploratory factor analysis (EFA) stage, and data from 366 participants were used for the confirmatory factor analysis (CFA) stage in the development of the organizational healing scale. The two factors (Individual Priority and Institutional Priority) identified after the EFA were confirmed through CFA.

### 2.2 Scale development process

Scale development studies should answer questions such as what is the construct to be measured, to whom will the scale be applied, for what purpose will the scores obtained from the scale be used, and what is the format of the scale items (Lane et al., 2016). Balcı (2018) generally states that the steps in the scale development process include creating an item pool, obtaining expert opinion, conducting a pilot study, and calculating validity and reliability.

As posited by Johnson and Morgan (2016), the development of scales is a common methodology employed by researchers to quantify the knowledge levels, behaviors, or perceptions of participants. The feature measured by the organizational healing scale was identified as the perceptions of the participants. The item pool for the organizational healing scale was developed in accordance with the principle of applicability for researchers and participants (DeVellis, 2017). In the preparation of the item pool, studies in the related field were taken into consideration (Powley, 2012, 2013; Powley and Piderit, 2008; Powley and Cameron, 2006). In this context, an item pool of 32 items was created. It is anticipated that the comprehensibility of the items in the item pool will be examined through the conduct of a technical audit regarding the adequacy of the items in measuring the targeted feature (Lane et al., 2016). In accordance with the aforementioned criteria, 32 items were presented to two experts in the field of educational administration and one expert in the field of Turkish language teaching. Additionally, the experts were requested to evaluate the scale items in terms of comprehensibility and scope. The 32-item scale was administered to a total of 465 individuals. The data set was subsequently filtered to exclude 24 responses that were identified as having been completed in an uncareful manner. In the absence of data, a value was assigned through the process of average assignment. The data obtained from 441 individuals were thus included in the subsequent validity and reliability analysis. Factor analysis can be divided into two categories: exploratory factor analysis (EFA) and confirmatory factor analysis (CFA) (Wang and Wang, 2020). To guarantee the accuracy and reliability of the findings, both EFA and CFA were employed to assess the construct validity, and the opinions of experts were sought to ensure content and face validity. The Cronbach alpha coefficient was calculated to ascertain the reliability of the data.

As stated by Tabachnick and Fidell (2014), kurtosis and skewness values serve as indicators of univariate normal distribution. The objective was to ascertain whether the data exhibited univariate normal distribution, a conclusion reached through an analysis of the skewness and kurtosis values. Upon examination of Table 2, it was determined that the skewness and kurtosis values of the study fall within the range of ±2, which is indicative of a normal distribution (George and Mallery, 2010).

**Table 2:** Descriptive statistics of the data set before EFA.

**Item no 1**	***n***	** $\bar{X}$ **	**sd**	**Skewness**	**Kurtosis**
Item no 2	441	3.40	1.185	−0.392	−0.802
Item no 3	441	3.55	1.054	−0.702	−0.141
Item no 4	441	3.75	1.110	−0.736	−0.260
Item no 5	441	3.71	1.016	−0.659	0.024
Item no 6	441	3.63	1.095	−0.543	−0.411
Item no 7	441	3.76	1.025	−0.840	0.343
Item no 8	441	3.85	0.990	−0.865	0.387
Item no 9	441	3.81	1.017	−0.859	0.338
Item no 10	441	3.59	1.125	−0.609	−0.346
Item no 11	441	3.73	1.110	−0.674	−0.290
Item no 12	441	3.74	1.052	−0.752	0.068
Item no 13	441	3.19	1.093	−0.159	−0.724
Item no 14	441	3.82	0.906	−0.709	0.336
Item no 15	441	3.70	0.976	−0.619	0.047
Item no 16	441	3.73	1.011	−0.801	0.310
Item no 17	441	3.84	0.960	−0.880	0.662
Item no 18	441	3.86	0.944	−0.899	0.699
Item no 19	441	3.20	1.079	−0.192	−0.610
Item no 20	441	3.76	0.941	−0.892	0.834
Item no 21	441	3.93	0.824	−1.110	1.932
Item no 22	441	3.71	1.018	−0.658	0.002
Item no 23	441	3.62	1.081	−0.560	−0.294
Item no 24	441	3.56	1.077	−0.612	−0.140
Item no 25	441	3.47	1.081	−0.361	−0.500
Item no 26	441	3.56	1.030	−0.633	−0.058
Item no 27	441	3.72	0.988	−0.828	0.525
Item no 28	441	3.39	1.098	−0.441	−0.434
Item no 29	441	3.49	1.053	−0.547	−0.319
Item no 30	441	3.72	0.983	−0.747	0.251
Item no 31	441	3.67	0.988	−0.675	0.034
Item no 32	441	3.57	1.036	−0.655	−0.072

As Carpenter (2018) asserts, it is imperative to ascertain the suitability of the sample for factorization through the utilization of Bartlett's Test of Sphericity and Kaiser-Meyer-Olkin (KMO) Test prior to the execution of Exploratory Factor Analysis. Field (2018) posits that the outcome of Bartlett's Test of Sphericity should be statistically significant and that the KMO value should exceed 0.50. In the present study, the results of Bartlett's test of sphericity were found to be statistically significant (χ^2^ = 11,093, *p* = 0.000) and the KMO value was 0.97.

Reckelkamm et al. (2023) demonstrate the efficacy of bootstrapping in mimicking new patient data to assess model quality, thereby circumventing the stringent requirement of multivariate normality. The coefficients for the direct effects, as determined using a 95% confidence interval, can be seen in Table 3.

**Table 3:** Bootstrapping for the model.

	**Coefficients**	**Lower bound**	**Upper bound**
Item no 1	0.626^**^	0.564	
Item no 2	0.746^**^	0.694	
Item no 3	0.865^**^	0.833	
Item no 4	0.872^**^	0.84	
Item no 5	0.829^**^	0.785	
Item no 6	0.858^**^	0.827	
Item no 7	0.81^**^	0.768	
Item no 10	0.769^**^	0.718	
Item no 16	0.663^**^		0.599
Item no 17	0.696^**^		0.63
Item no 18	0.546^**^		0.48
Item no 23	0.756^**^		0.709
Item no 24	0.808^**^		0.766
Item no 25	0.811^**^		0.769
Item no 26	0.809^**^		0.768
Item no 27	0.727^**^		0.675
Item no 28	0.858^**^		0.823
Item no 29	0.88^**^		0.854
Item no 30	0.841^**^		0.808
Item no 31	0.813^**^		0.766
Item no 32	0.842^**^		0.8

^**^p < 0.01.

## 3 Findings

The scale was developed in line with the study's purpose, with particular emphasis placed on construct, content, and face validity.

### 3.1 Construct validity

In order to ensure construct validity of the scale developed within the scope of the study, an exploratory factor analysis (EFA) was conducted initially, followed by a confirmatory factor analysis (CFA).

#### 3.1.1 EFA findings

After testing the suitability of the data for EFA, principal component analysis was applied to the data, and the Varimax vertical rotation method was used. As a result of principal component analysis, two factors with eigenvalues above 1 emerged.

An examination of the rotated components matrix revealed that the highest factor loadings were concentrated in the initial two factors. In accordance with the stipulations set forth by Johnson and Morgan (2016), the factor loadings of the items pertaining to a given factor were deemed to be acceptable if they reached a minimum value of 0.40. Consequently, this criterion was applied in the present study, with factor loading values determined to be at least 0.40. Furthermore, if items with factor loadings above 0.40 are classified in multiple factors and the differences between these values are below 0.20, the items should be excluded from the scale, as they exhibit overlap (Child, 2006). In this study, after the removal of 11 items due to low factor loadings and overlap, the analysis was conducted using the varimax vertical rotation method. The results are presented in Table 4.

**Table 4:** Eigenvalues of the organizational healing scale and variance ratio explained by factors.

**Components**	**Initial eigenvalues**			**Rotation sums of squared loadings**		
	**Total**	**% of variance**	**Cumulative %**	**Total**	**% of variance**	**Cumulative %**
1	11.283	53.727	53.727	7.125	33.929	33.929
2	1.518	7.226	60.953	5.675	27.024	60.953
3	0.961	4.577	65.530			
4	0.818	3.893	69.423			
5	0.727	3.460	72.883			
6	0.694	3.303	76.186			
7	0.568	2.703	78.889			
8	0.511	2.435	81.324			
9	0.485	2.311	83.635			
10	0.441	2.101	85.736			
11	0.389	1.852	87.588			
12	0.367	1.750	89.338			
13	0.335	1.593	90.931			
14	0.309	1.471	92.402			
15	0.290	1.380	93.782			
16	0.261	1.245	95.027			
17	0.244	1.163	96.190			
18	0.230	1.094	97.284			
19	0.200	0.955	98.238			
20	0.196	0.933	99.172			
21	0.174	0.828	100.000			
Extraction Method: Principal Component Analysis						

The Table 4 presents the eigenvalues associated with the factor structure of the Organizational healing Scale and the variance ratios explained by the factors. The total variance ratio of the scale, which was determined to be two factors, was determined to be 60.9%. Accordingly, the contribution of the first factor to the total variance was 33.9%, and the contribution of the second factor to the total variance was 27%. It is recommended that the variance ratio obtained through factor analysis should ideally fall between 40% and 60% (Scherer et al., 1988). In this instance, the variance ratio obtained is deemed to be at an adequate level. Subsequently, Table 5 illustrates the categorization of the items in accordance with the identified factors.

**Table 5:** Rotated component matrix after factor analysis of organizational healing scale.

**Items**	**Components**	
	**Individual priority**	**Organizational priority**
Item no 1		0.682
Item no 2		0.703
Item no 3		0.821
Item no 4		0.822
Item no 5		0.768
Item no 6		0.745
Item no 7		0.632
Item no 10		0.665
Item no 16	0.631	
Item no 17	0.660	
Item no 18	0.530	
Item no 23	0.707	
Item no 24	0.714	
Item no 25	0.660	
Item no 26	0.634	
Item no 27	0.649	
Item no 28	0.777	
Item no 29	0.739	
Item no 30	0.742	
Item no 31	0.728	
Item no 32	0.741	

The Table 5 illustrates the factors that inform the grouping of the items. In accordance with this categorization, the “Individual Priority” factor includes items M1, M2, M3, M4, M5, M6, M7, M10, whereas the “Organizational Priority” factor includes items M16, M17, M18, M23, M24, M25, M26, M27, M28, M29, M30, M31, M32.

Table 6 presents the findings pertaining to the components in which the scale items are grouped.

**Table 6:** Items and components of organizational healing scale.

**Component**	**Item no**	**Item**
Individual priority	Item no 1	After crises or traumatic events, members of the organization avoid blaming each other.
	Item no 2	Members of the organization are enabled to quickly confront crises or traumatic events.
	Item no 3	After crises and traumatic events, management strives to restore members of the organization to their previous wellbeing.
	Item no 4	After crises or traumatic events, management acts with the future of the members of the organization in mind.
	Item no 5	After crises or traumatic events, the management pays attention to the careers of the members of the organization.
	Item no 6	In the aftermath of crises or traumatic events, management prioritizes the care of members in urgent need.
	Item no 7	After crises or traumatic events, the management meets with the members who have been adversely affected.
	Item no 10	Communication between members of the organization is based on trust.
Organizational priority	Item no 16	To prevent crises or traumatic events from happening again, negative experiences are commemorated with regular events.
	Item no 17	After crises or traumatic events, activities are organized to refocus the organization on its goals.
	Item no 18	Necessary measures are taken against possible new crisis situations after crises or traumatic events.
	Item no 23	All members of the organization learn from crises or traumatic events.
	Item no 24	My organization emerges stronger from crises or traumatic events.
	Item no 25	After crises or traumatic events, the management develops scenarios for possible crisis situations.
	Item no 26	Shared experiences from crises or traumatic events strengthen the organization.
	Item no 27	In order to prevent crises or traumatic events from happening again, negative experiences are honored with regular events.
	Item no 28	After crises or traumatic events, activities are organized to refocus the organization on its goals.
	Item no 29	Necessary measures are taken against possible new crisis situations after crises or traumatic events.
	Item no 30	After crises or traumatic events, my organization makes changes in routine actions or arrangements.
	Item no 31	Anxiety-reducing changes for crises and traumatic events are determined by the manager of the organization.
	Item no 32	Changes to prevent crises and traumatic events are initiated by the manager of the organization.

Upon examination of the items comprising the factors, the first component, consisting of eight items, was designated as “Individual Priority,” while the second component, comprising 13 items, was designated as “Organizational Priority.” Upon examination of the entire Organizational healing Scale, it becomes evident that it comprises 21 items and two components.

#### 3.1.2 CFA findings

In order to confirm the 2-factor and 21-item scale structure that emerged as a result of EFA, the CFA application was conducted as the other stage of the validity of the Organizational Healing scale. To prevent erroneous results in testing the scale structure, it is recommended that CFA be conducted with data obtained from a new sample, as opposed to EFA (Henson and Roberts, 2006). In this context, the scale completed by 384 participants was reduced to 366 participants after outliers were removed from the data set. It has been suggested that a sample size of more than 300 participants would be sufficient for a CFA application (Worthington and Whittaker, 2006). The findings obtained as a result of the CFA conducted to confirm the two-factor structure of the organizational healing scale are presented in Table 7.

**Table 7:** Confirmatory factor analysis fit indices of organizational healing scale.

**Fit indices**	**Indices relevant to the model**	**Excellent fit values**	**Acceptable fit values**
*X* ^2^	567.8		
df	185		
*X*^2^/df	3	0 ≤ *X*^2^/sd ≤ 2	2 ≤ *X*^2^/sd ≤ 3
RMSEA	0.075	0 ≤ RMSEA ≤ 0.05	0.05 ≤ RMSEA ≤ 0.08
SRMR	0.03	0 ≤ SRMR ≤ 0.05	0.05 ≤ SRMR ≤ 0.10
NFI	0.92	0.95 ≤ NFI ≤ 1.00	0.90 ≤ NFI ≤ 0.95
NNFI	0.93	0.97 ≤ NNFI ≤ 1.00	0.90 ≤ NNFI < 0.97
CFI	0.94	0.97 ≤ CFI ≤ 1.00	0.95 ≤ CFI ≤ 0.97
GFI	0.88	0.95 ≤ GFI ≤ 1.00	0.80 ≤ GFI < 0.95
AGFI	0.84	0.90 ≤ AGFI ≤ 1.00	0.80 ≤ AGFI ≤ 0.90

Kline (2015, p. 269) posits that a variety of fit indices should be evaluated through the application of CFA, with the objective of verifying the model that emerges from EFA. The model fit indices and the reference intervals for the perfect and acceptable fit indices for the organizational healing scale are provided in Table 7 (Büyüköztürk et al., 2020; Schermelleh-Engel et al., 2003; Hu and Bentler, 1999; Schumacker and Lomax, 2016).

**Figure 2:**
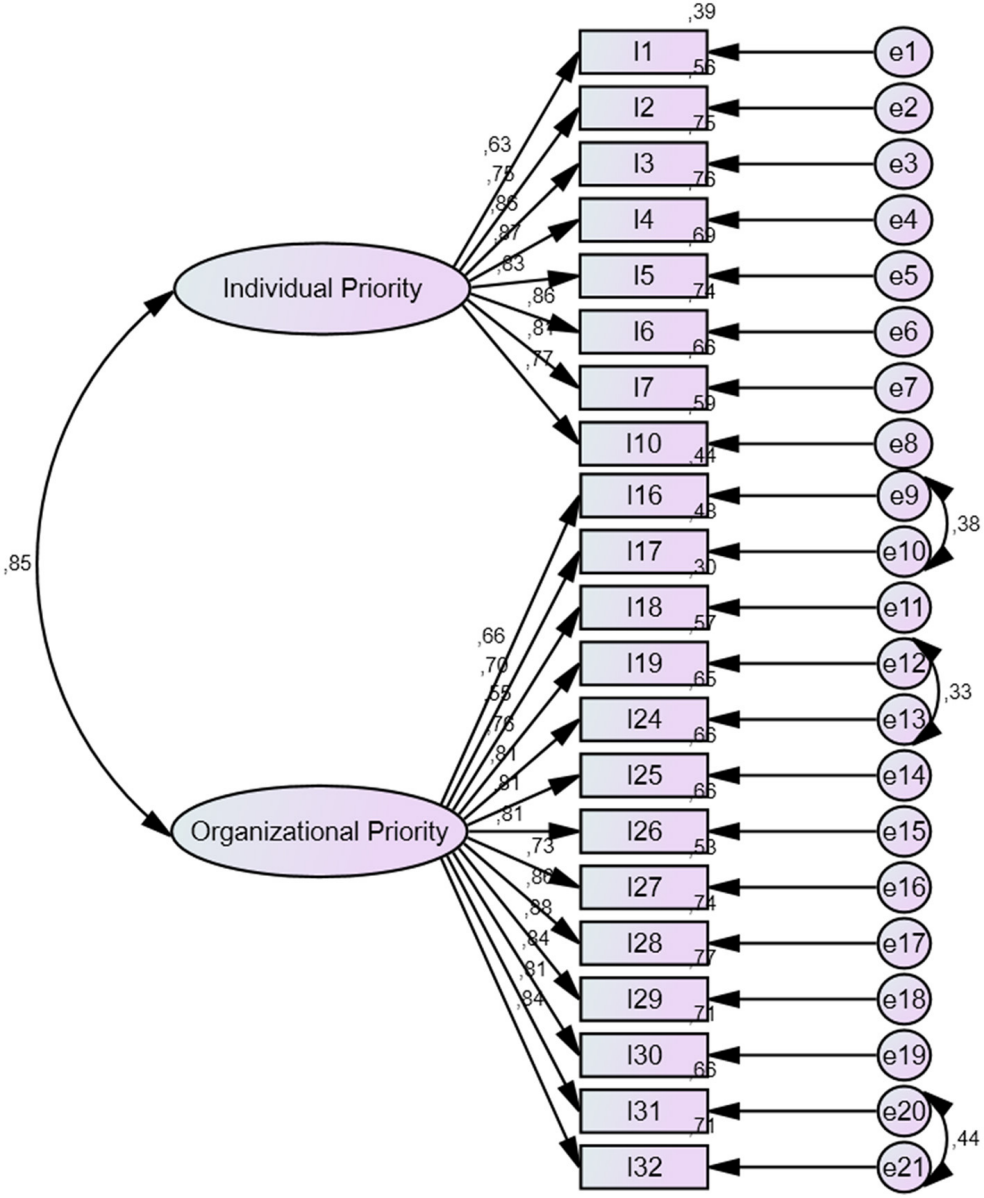
Confirmatory factor analysis diagram of the organizational healing scale.

As a consequence of the CFA, the value of χ^2^ was found to be 567.8, with a degrees of freedom value of 185. The *p*-value was determined to be 0.000, which is below the threshold of 0.05 (Hooper et al., 2008). Additionally, the RMSEA value was found to be below 0.08 (Schermelleh-Engel et al., 2003), The SRMR value was found to be below 0.08 (Hu and Bentler, 1999), the GFI value should be above 0.80 (Hu and Bentler, 1999), the AGFI value should be above 0.80 (Segars and Grover, 1993), and the NFI value should be above 0.80 (Segars and Grover, 1993). In the present study, the NFI value is above 0.80 (Hu and Bentler, 1999), the NNFI (TLI) value is over 0.90 (Hu and Bentler, 1999), and the CFI value is 0.94 (Kline, 2015), indicating that the scale model proposed is validated. The model obtained through the CFA conducted for the Organizational Healing scale is presented in Figure 2.

Upon examination of the Figure 2, it becomes evident that the organizational healing scale is comprised of two components: “Individual Priority” and “Organizational Priority.” It is observed that the factor loadings of the model for the organizational healing scale exhibit a range of values between 0.55 and 0.88.

An additional method for determining item discrimination is to examine whether there is a significant relationship between the high and low scores obtained from the relevant item. The use of 27% score intervals for the lower and upper groups is a preferred approach in terms of ease of comprehension and interpretability (DeMar, 2018). The statistical values for the item discrimination of the organizational healing scale are presented in Table 8.

**Table 8:** The scores of the participants in the lower and upper 27% for the organizational healing scale.

**Groups**	**27% score intervals for the lower groups**		**27% score intervals for the upper groups**		***t (df)***	***p***
	$\bar{X}$	**sd**	$\bar{X}$	**sd**		
Item 1	2.76	0.975	4.46	0.735	13.82 (194)	0.000
Item 2	2.85	0.901	4.46	0.595	14.78 (194)	0.000
Item 3	2.85	0.934	4.79	0.460	18.42 (194)	0.000
Item 4	2.88	0.828	4.62	0.566	17.22 (194)	0.000
Item 5	2.72	0.894	4.63	0.616	17.39 (194)	0.000
Item 6	2.96	0.884	4.71	0.497	17.12 (194)	0.000
Item 7	3.05	1.009	4.79	0.412	15.75 (194)	0.000
Item 8	2.80	0.896	4.71	0.556	18.00 (194)	0.000
Item 9	3.24	0.862	4.66	0.591	13.43 (194)	0.000
Item 10	3.24	0.909	4.61	0.549	12.74 (194)	0.000
Item 11	2.66	0.963	4.10	0.891	10.85 (194)	0.000
Item 12	2.76	0.897	4.50	0.630	15.75 (194)	0.000
Item 13	2.72	0.757	4.55	0.644	18.19 (194)	0.000
Item 14	2.82	0.817	4.50	0.579	16.65 (194)	0.000
Item 15	2.99	0.855	4.58	0.555	15.45 (194)	0.000
Item 16	2.62	0.856	4.28	0.743	14.43 (194)	0.000
Item 17	2.63	0.842	4.53	0.596	18.21 (194)	0.000
Item 18	2.83	0.874	4.69	0.485	18.49 (194)	0.000
Item 19	2.87	0.820	4.60	0.492	17.95 (194)	0.000
Item 20	2.82	0.854	4.51	0.613	15.95 (194)	0.000
Item 21	2.95	0.866	4.72	0.471	17.82 (194)	0.000

Kelley (1939) emphasizes the importance of focusing on the upper and lower 27% groups when analyzing test items. This method allows researchers to concentrate on the extremes of the data distribution. As seen in Table 8, the items on the scale have the capacity to distinguish between high- and low-performing individuals in a meaningful way, which is essential to validate the scale.

In order to ascertain the reliability of the structure that emerged as a result of the exploratory factor analysis (EFA) and confirmatory factor analysis (CFA) conducted for the Organizational healing Scale, a Cronbach's Alpha (α) coefficient was calculated. The resulting Cronbach Alpha (α) coefficients are presented in Table 9.

**Table 9:** Cronbach alpha (α) reliability coefficients of the organizational healing scale.

**Components**	**Cronbach alpha (α)**
Individual priority	0.93
Organizational priority	0.95

A Cronbach alpha (α) internal consistency coefficient value of 0.90 or above is indicative of a very high level of reliability (Kline, 2015, p. 92). In this context, analysis of the internal consistency coefficients indicate that individual priority and organizational priority components are reliable.

### 3.2 Convergent and discriminant validity

In addition to EFA and CFA, convergent and discriminant validity studies were also conducted to examine the construct validity of the Organizational Healing Scale. The high factor loadings obtained from the CFA indicate that the scale achieved convergent validity. In addition to factor loadings, Average Variance Extracted (AVE) values can be examined to determine whether convergent validity is achieved. An AVE above 0.50 is considered evidence of convergent validity (Fornell and Larcker, 1981). Table 10 shows the AVE values for the factor loadings obtained from CFA.

**Table 10:** AVE values for factor loadings.

**Components**	**AVE values**
Individual priority	0.58
Organizational priority	0.61

Upon examination of the findings presented in Table 10, it becomes evident that the AVE values calculated for the factor loadings obtained from CFA exceed the 0.50 criterion (Shrestha, 2021). Consequently, it can be asserted that the Organizational Healing Scale has met the requisite criteria for convergent validity.

In examining the discriminant validity, it is necessary to ensure that the value obtained by taking the square root of the AVE for each dimension is greater than the correlation between the dimensions and above 0.50 (Fornell and Larcker, 1981). Table 11 presents discriminant validity of the scale of the organizational healing scale.

**Table 11:** Discriminant validity of the scale.

**Components**	**Individual priority**	**Organizational priority**
Individual priority	1	
Organizational priority	0.57	1

Upon examination of the findings presented in Table 11, it becomes evident that the square root AVE value calculated for each dimension is higher than the correlation between the subscales, exceeding the criterion of 0.50. These findings substantiate the assertion that the Organizational Healing Scale has achieved discriminant validity.

The teachers' professional resilience scale was used to calculate organizational healing construct for the criterion-related validity. Given the significant differences in the individual and organizational priority means for overall resilience, as evidenced by the correlation analyses in Table 12, it can be concluded that both the model (individual priority: *F* = 266.81, *p* < 0.001, *R*^2^: 0.52; organizational priority: *F* = 89.61, *p* < 0.001, *R*^2^: 0.26) and the construct demonstrates criterion validity (Bollen, 1989).

**Table 12:** Criterion-related validity of the scale.

**Components**	**Individual priority**	**Organizational priority**	**Professional resilience**
Individual priority	1	0.765^**^	0.721^**^
Organizational priority		1	0.516^**^
Professional resilience			1

^**^p < 0.01.

## 4 Discussion

The objective of this study is to develop an organizational healing scale. In the course of this study, two components were identified: “individual priority” and “organizational priority.” The individual priority factor consists of eight items, while the organizational priority factor consists of 13 items. In the preparation of these items, the organizational healing literature was consulted, as well as related literature. The concept of healing is inherently linked to the occurrence of harm or damage. It is inaccurate to assume that healing occurs when circumstances are favorable. Consequently, it is challenging to ascertain the extent to which an organization is capable of recovering from a traumatic event, as this cannot be determined until the incident has occurred. However, through the use of simulated or actual traumas, organizational members can gain insight into their organization's capacity to restore itself to a state of health and integrity, which can be defined as its level of healing (Powley and Cameron, 2006). The process and mechanisms of organizational healing represent an important aspect for improving organizations in the face of challenges and harm. When healing mechanisms are in place, organizational healing represents a process for strengthening relationships, activating positive outcomes and restoring organizations to positive health. Organizational healing relies on known concepts to suggest not only a return to normal routines and previous states, but also a process of growth where healing provides organizational strength. Healing provides organizational strength through positive practices, collective action, leadership activities and associated structures and routines (Powley, 2012). In this respect, it is considered very important to know the organizational healing levels of organizations and in this study, an “Organizational Healing Scale” was developed to fill this gap in the field.

A total of 441 individuals participated in the exploratory factor analysis (EFA) phase, while 366 individuals participated in the confirmatory factor analysis (CFA) phase. The KMO test was conducted to ascertain the suitability of the scale for factorization. The two-factor variances of the organizational healing scale indicated that the scale has sufficient values (Hair et al., 2018). Upon examination of the fit indices associated with the organizational healing scale, it becomes evident that the obtained values indicate a good to excellent fit level. In order to validate the scale model, it is necessary to ensure χ^2^/sd value (Hu and Bentler, 1999), RMSEA value (Schermelleh-Engel et al., 2003), SRMR value (Hu and Bentler, 1999), GFI value (Hu and Bentler, 1999), AGFI value (Segars and Grover, 1993), NFI value (Hu and Bentler, 1999), NNFI value (Hu and Bentler, 1999) and CFI value (Kline, 2015). In light of the evidence presented, it can be concluded that the scale model depicted in Figure 2 is an empirically valid representation of the underlying construct.

According to Kline (2015, p. 92), values of 0.90 and above indicate very high reliability for the Cronbach Alpha internal consistency coefficient. Upon examination of the Cronbach Alpha coefficient of the factors and the total scale, it was determined that the organizational healing scale exhibited high reliability. As evidenced by the rotated components matrix obtained following the exploratory factor analysis (EFA) conducted to determine the factor loadings of the organizational healing scale, the lower limit of factor loading estimation was set at 0.40. Upon analysis of the rotated components matrix of the scale, it was observed that the factor loadings obtained were of a notably high level (Costello and Osborne, 2005; Tabachnick and Fidell, 2014).

The Organizational Healing Scale can assist in determining the status of an organization's return to normal functioning and enhancing its operational efficacy following adverse circumstances that impair the organizational structure and interpersonal relationships. Furthermore, the Organizational Healing Scale can be utilized to ascertain the influence of various interventions on the organizational context and to identify potential avenues for improvement. An examination of analogous studies on this subject reveals that the majority of them (Doganay and Dagli, 2020; Singh and Jha, 2018) address organizational trauma, organizational health, and related matters. Accordingly, the scale developed in this research will contribute to the existing literature on this topic by providing a tool for assessing the potential for organizational healing following a adverse event.

## Data Availability

The raw data supporting the conclusions of this article will be made available by the authors, without undue reservation.
